# Target product profiles for protecting against outdoor malaria transmission

**DOI:** 10.1186/1475-2875-11-17

**Published:** 2012-01-11

**Authors:** Gerry F Killeen, Sarah J Moore

**Affiliations:** 1 Biomedical and Environmental Thematic Group, Ifakara Health Institute, PO Box 53, Ifakara, Morogoro, United Republic of Tanzania; 2 Vector Group, Liverpool School of Tropical Medicine, Pembroke Place, Liverpool L3 5QA, UK; 3 Department of Disease Control, London School of Hygiene and Tropical Medicine, Keppel Street, London WC1E 7HT, UK

**Keywords:** integrated vector management, pesticide, *Anopheles*, *Plasmodium*, transmission dynamics

## Abstract

**Background:**

Long-lasting insecticidal nets (LLINs) and indoor residual sprays (IRS) have decimated malaria transmission by killing indoor-feeding mosquitoes. However, complete elimination of malaria transmission with these proven methods is confounded by vectors that evade pesticide contact by feeding outdoors.

**Methods:**

For any assumed level of indoor coverage and personal protective efficacy with insecticidal products, process-explicit malaria transmission models suggest that insecticides that repel mosquitoes will achieve less impact upon transmission than those that kill them outright. Here such models are extended to explore how outdoor use of products containing either contact toxins or spatial repellents might augment or attenuate impact of high indoor coverage of LLINs relying primarily upon contact toxicity.

**Results:**

LLIN impact could be dramatically enhanced by high coverage with spatial repellents conferring near-complete personal protection, but only if combined indoor use of both measures can be avoided where vectors persist that prefer feeding indoors upon humans. While very high levels of coverage and efficacy will be required for spatial repellents to substantially augment the impact of LLINs or IRS, these ambitious targets may well be at least as practically achievable as the lower requirements for equivalent impact using contact insecticides.

**Conclusions:**

Vapour-phase repellents may be more acceptable, practical and effective than contact insecticides for preventing outdoor malaria transmission because they need not be applied to skin or clothing and may protect multiple occupants of spaces outside of treatable structures such as nets or houses.

## Background

Long-lasting insecticidal nets (LLINs) and indoor residual spraying (IRS) have dramatically reduced malaria transmission by indoor-feeding (endophagic) mosquito populations in recent years [1-4]. However, elimination of transmission is not currently considered possible without cost-effective new technologies that protect against the persistent outdoor-biting (exophagic) vectors that continue to mediate self-sustaining residual transmission [4-6] because they are less vulnerable to insecticides applied to indoor surfaces [7-9]. Now that LLINs and IRS are being successfully scaled up in many countries across the tropics [10], it is timely to consider the potential of complementary products capable of protecting humans against these residual vector populations while outside of their houses.

While mosquitocidal vaccines [11] and drugs [12] offer the exciting possibility of around-the-clock protection, wherever and whenever users are exposed, these products require systemic administration to humans and are in relatively early stages of development and evaluation as malaria vector control tools. In contrast, the most immediately available options simply extend coverage of safe, widely-used pyrethroid insecticides beyond the house by formulating them as treatments for clothing [13,14]. Alternatively, some fluorinated pyrethroids are far more volatile than the active ingredients currently used for LLINs and IRS so that emanators can deliver them to protected spaces in vapour phase, even where no treatable surface exists [15,16].

However, it is crucial to clearly distinguish between alternative modes of action of vector control products and assess their potential comparative value for preventing malaria. Pesticide products either deter insects away from protected houses, sleeping spaces and humans, or they kill those that make physical contact with them [17]. While repellency or deterrence obviously enhances personal or household protection by LLINs or IRS, recently developed process-explicit models [8,18] support field observations [19-21] that it may also attenuate the even greater community-wide protection that can be achieved by high coverage of products with contact toxicity [22,23]. This is because mosquitoes are not killed outright and may therefore be diverted to nearby community members, some of whom are unprotected non-users [8]. It is, therefore, essential to consider the subtle mechanisms of action of spatial repellents, and their potential practicality, when assessing how much control of outdoor malaria transmission could be achieved in comparison with mosquito-toxic insecticides [13,14], vaccines [11] or drugs [12] that directly kill mosquitoes but require physical contact with the human user.

## Methods

Existing models of the interaction between contrasting deterrent and toxic properties [8,18] assume that these are combined in a single protected indoor space. Here these models [8] are extended to consider products with the following profiles used singly or in combination: 1) an LLIN conferring contact fast-acting toxicity that can only be used indoors, 2) an equivalent contact toxic product that can be only be used outdoors, 3) a spatial repellent product that is exclusively used outdoors or 4) a spatial repellent that is used both indoors and outdoors. The impact of these measures was expressed in terms of the relative risk of exposure to transmission (ψ), calculated as the predicted entomological inoculation rate (EIR) experienced by humans (h) in a given scenario (Ω) of coverage and efficacy for these measures (EIR_h,Ω_) compared with that predicted under baseline conditions (EIR_h,0_) where neither measure was in place:

$$\psi_{\Omega }=\text{EI}{\text{R}}_{\text{h,}\Omega }/\text{EI}{\text{R}}_{\text{h,}0}$$

**Figure 1:**
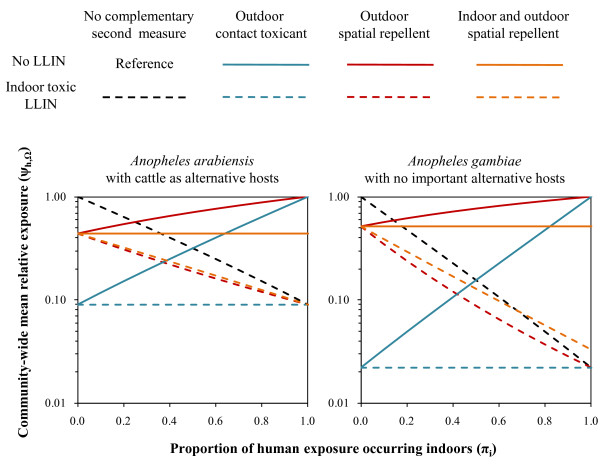
**Impact of products for outdoor malaria prevention expressed in terms of the mean relative risk of exposure experienced by the average community member (*ψ*_h,Ω_)**. Scenarios are considered in which LLIN products that provide 50% personal protection (ρ_i _= 0·5) by killing half of all mosquitoes that attack them (θ_μ,pre,i _= 0·5) are complemented by use of additional products conferring equivalent personal protection (ρ_o _or ρ_i+o _= 0·5) with one of the three following profiles: Products for exclusively outdoor use that kill attacking mosquitoes before they feed (θ_μ,pre,o _= 0·5) or products that deter mosquitoes from attacking that are used either outdoors only (θ_Δ,o _= 0·5) or are used both indoors and outdoors (θ_Δ,i+o _= 0·5). Further details of the model and symbol definitions are available online (See Additional file 4)

**Figure 2:**
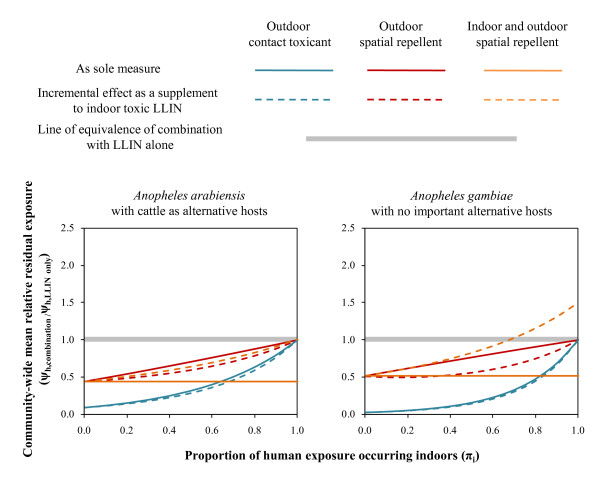
**Additional incremental impact of outdoor contact toxins (θ_μ,pre,o _= 0·5) or repellents that are exclusively used outdoors (θ_Δ,o _= 0·5) or used both indoors and outdoors (θ_Δ,i+o _= 0·5) when combined with indoor LLINs with contact toxins (θ_μ,pre__,i_ = 0·5), compared with their direct impact as stand-alone intervention strategies**. Impact is expressed in terms of the mean relative risk of exposure to residual transmission for the average community member where LLINs are combined with additional products with the above profiles (ψ_h,combination_) compared with when they are applied as a stand-alone measure (ψ_h,LLINs alone_). All products are assumed to confer 50% personal protection (ρ_o _or ρ_i+o _= 0·5) by either repelling or killing half of all mosquitoes that attack them (θ = 0·5). Further details of the model and symbol definitions are available online (See Additional file 4)

**Figure 3:**
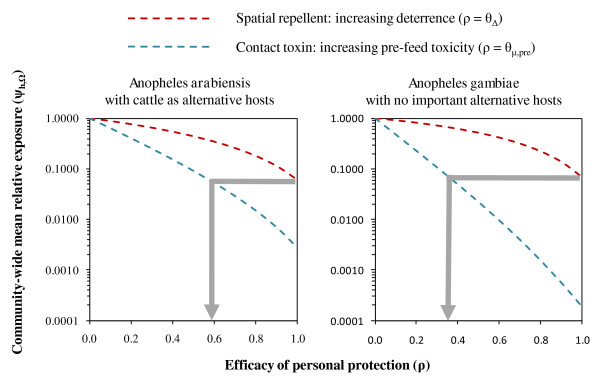
**Progressive impact upon a completely outdoor transmission system (π_i _= 0) of products with increasing efficacy of personal protection (ρ_o_) achieved by either repelling (θ_Δ,o_) or killing (θ_μ,pre,o_) attacking mosquitoes before they feed upon human users**. The grey arrows represent interpolation of the efficacy thresholds at which the toxic mode of action achieves equivalent transmission control to the theoretical limit at complete protective efficacy (θ_Δ,o _= 1·0 so ρ_o _= 1·0) for spatial repellents at high coverage (C_h _= 0·8). Impact is expressed in terms of the mean relative risk of exposure experienced by the average community member (ψ_h,_Ω). Further details of the model and symbol definitions are available online (See Additional file 4)

This term was calculated for unprotected individuals lacking either of these measures (ψ_h,0,Ω_) or as a community-wide average, reflecting the coverage-weighted mean of such non-users and users of one or both measures (ψ_h,Ω_) [8]. For simplicity, only the community-wide average relative exposure and relative residual exposure for each scenario, reflecting combined community-level and personal protection effects, are presented in the main text in Figures 1, 2, 3). For comparison, equivalent plots of relative exposure and relative residual exposure of non-users, reflecting community-level protection effects only, are available online (See Additional file 1, Additional file 2, and Additional file 3).

In all cases, coverage levels of 80% were assumed for both the indoor and outdoor protection measures, consistent with current global targets for LLINs and IRS [24], and two distinct Afrotropical vector population scenarios were examined: strongly anthropophagic *Anopheles gambiae*, which rarely uses cattle as alternative non-human hosts, and zoophagic *Anopheles arabiensis*, which does where they are available. These relative exposure outcomes (ψ) were predicted as a function of the mean proportion of malaria transmission exposure of a non-user of any measure which occurs indoors (π_i_) [9,25,26] and the personal protective efficacy (ρ) arising from increased deterrence (θ_Δ_) or pre-feed mortality (θ_μ,pre_) of mosquitoes attacking users of the product [8]. While this model can just as readily simulate the impact of IRS, only LLINs were considered as a potential means of indoor protection because the combination of the physical barrier or the net and the fast-acting toxicity of their pyrethroid active ingredients allow them to be directly compared with spatial repellents or insecticidal clothing that also confer direct personal or household protection [27]. By comparison, many IRS formulations are relatively slow-acting, usually killing mosquitoes after they have fed, so that the comparison between repellent and toxic modes of action is confounded by the differences between slow and fast-acting toxins [8,27]. Therefore, no scenarios including IRS as the indoor protection measure were simulated. Further details of the extended model and its application in this report are available online (See Additional file 4).

## Results

LLINs depend upon high proportions of human exposure occurring indoors to achieve maximum impact upon malaria transmission (Figure 1). Note that the apparent lack of impact of indoor LLIN use where mosquitoes prefer to feed outdoors should be interpreted cautiously. It is critical to determine whether these behavioural characteristics reflect historical baseline values consistent with those observed before these indoor control measures were introduced, rather than modified transmission patterns associated with residual vector populations after intervention scale up [4-6]. Indoor use of such insecticidal products may have little value where no major indoor-biting vectors have historically existed or where they have been successfully eliminated. However, contemporary observations of high proportions of outdoor exposure can simply reflect successful suppression of previously abundant endophagic populations [4-6,28-31], that can readily recover and restore high transmission levels if coverage with LLINs is not sustained.

Exclusive outdoor use of products that either kill or repel mosquitoes consistently complements LLINs (Figure 1) by filling the protective coverage gap that occurs in the rural tropics where people commonly spend their evenings outside. In stark contrast with the effects of supplementing toxicity with repellency within a single product applied in the same place [8], where at least half of transmission occur outdoors (0·5 ≤ π_i_), spatial repellents applied outdoors consistently supplement the impact of indoor toxins (Figure 2). In fact, spatial repellents even synergize with LLINs for anthropophagic vectors (Figure 2), with an optimum where transmission occurs both indoors and outdoors (0·2 < π_i _< 0·8) because mosquitoes diverted from outdoor repellent users may subsequently attack those protected indoors by lethal LLINs (See Additional file 1 and Additional file 2). Conversely, if repellents are also used indoors in settings where LLINs are common, mosquitoes can be deterred from exposure to fatal contact with products so that overall protection is attenuated where most transmission occurs indoors (π_i _> 0·7) and vectors rarely feed upon non-human blood sources (Figures 1 and 2). Such conditions would necessitate the avoidance of combined indoor use of spatial repellents alongside LLINs for the former to augment the communal protection already achieved by the latter. This caveat even applies to settings where residual transmission following LLINs scale-up is dominated by exophagic or zoophagic mosquitoes [1,4-6]. Except in unusual cases where they have been irreversibly eliminated from isolated islands [4], historically important endophagic and anthropophagic mosquito populations may well recover if spatial repellents are introduced to houses already using LLINs so that sub-lethal exposure to the former undermines the degree of lethal exposure to the latter.

Contact toxins appear superior to spatial repellents at any given level of coverage and personal protective efficacy (Figure 3). However, significant practical obstacles may render high efficacy and coverage targets more difficult to achieve with contact toxins applied to humans while outdoors. Protection of the entire vulnerable skin surface with either clothing or topical applications may be unrealistic in most tropical settings. Regular bathing, as well as washing and ironing of clothing, present significant barriers to high coverage with contact toxins in many societies. Furthermore, high coverage may be as difficult to achieve as durable high efficacy because user acceptance obviously depends on perceived personal protection. Subsequent simulations therefore examined and compared the relationship between impact and protective efficacy for these two distinct modes of action.

While spatial repellents are predicted to confer useful, but nevertheless limited levels of community-level protection (See Additional file 3), this may be complemented by high levels of personal protection to achieve far greater overall impact (Figure 3). Measures relying on contact toxins would need to kill between 36 and 56% of mosquitoes that attack protected users to match the expected benefits of high coverage with a spatial repellent conferring near-complete protective efficacy (Figure 3). Such levels of efficacy for contact insecticides are approximately equivalent to those observed for LLINs products that have been washed or undergone long periods of regular use [27] and may be more challenging to achieve in the absence of such a near-complete protective barrier as a net.

## Discussion

Despite their apparent theoretical inferiority to contact toxins, there are several practical reasons why spatial repellents, that protect a space rather than a surface [32] may be equally useful for preventing outdoor transmission of malaria and other mosquito-borne pathogens.

While contact irritants have no obvious practical advantage over contact toxins, vapour-phase repellents may be more acceptable and practical for outdoor use because they do not need to be applied to the skin or clothing of active humans when they are outside of treatable structures such as nets or houses. Pesticides that are volatile enough to diffuse through air negate the need to treat the skin or clothing and can protect a space surrounding a delivery point so that a single unit can protect multiple users for long periods [15] without requiring regular re-application or unrealistic levels of user compliance [33].

The hypothesis that outdoor malaria transmission can be controlled effectively with either contact insecticides or spatial repellents can only be tested through rigorous field evaluations of products that match the corresponding target product profiles suggested by these simulations. While vaccines [11] and drugs [12] that render human blood toxic to mosquitoes represent exciting possibilities for the future, permethrin-treated clothing [13,14] offers the closest existing approximation to the target product profile predicted for outdoor use of contact insecticides. For spatial repellents, it is encouraging that products matching the ambitious efficacy targets predicted here [15], exciting leads for new active ingredients [34,35], and creative delivery methods that restrict use to waking hours when people are usually outside [16], have all been described.

In summary, formulations of either contact insecticides or spatial repellents currently appear to have equal potential for preventing, or even eliminating, outdoor malaria transmission. Protection against indoor-biting malaria mosquitoes with LLINs or IRS is becoming a norm across growing tracts of the tropics. It is, therefore, time to prioritize development and field evaluation of new products designed specifically to tackle malaria transmission occurring outside of treatable structures such as walls, roofs or nets.

## Competing interests

While this study was independently funded by the Bill & Melinda Gates Foundation, both authors have received funding support for other research projects from manufactures of insecticidal public health products: Vestergaard Frandsen SA (GFK), Syngenta (SJM), Pinnacle Development (SJM) and SC Johnson (SJM).

## Authors' contributions

Both authors formulated the research questions and developed the conceptual basis of the model. GFK drafted the model formulation and manuscript, which was then critiqued and edited by SJM. All authors have read and approved the final version of the manuscript.

## Supplementary Material

Additional file 1 **Figure S1**. Purely community-level impact of products for outdoor malaria prevention expressed in terms of the mean relative risk of exposure experienced by non-users of any protective measure. Click here for file

Additional file 2 **Figure S2**. Additional incremental community-level impact of outdoor contact toxins or repellents that are exclusively used outdoors or used both indoors and outdoors when combined with indoor LLINs with contact toxins, compared with their direct impact as stand-alone intervention strategies. Click here for file

Additional file 3 **Figure S3**. Progressive community-level impact upon a completely outdoor transmission system of products with increasing efficacy of personal protection achieved by either repelling or killing attacking mosquitoes before they feed upon human users. Click here for file

Additional file 4 **Supplemental methods S4**. Detailed model description. Click here for file
