# Supplementary material for: Target product profiles for protecting against outdoor malaria transmission
Source: Malar J. 2012 Jan 11;11:17. doi: 10.1186/1475-2875-11-17 (PMC3298720; doi:10.1186/1475-2875-11-17)
Supplement: Additional file 1 — Figure S1. Purely community-level impact of products for outdoor malaria prevention expressed in terms of the mean relative risk of exposure experienced by non-users of any protective measure. [file 1475-2875-11-17-S1.PDF]

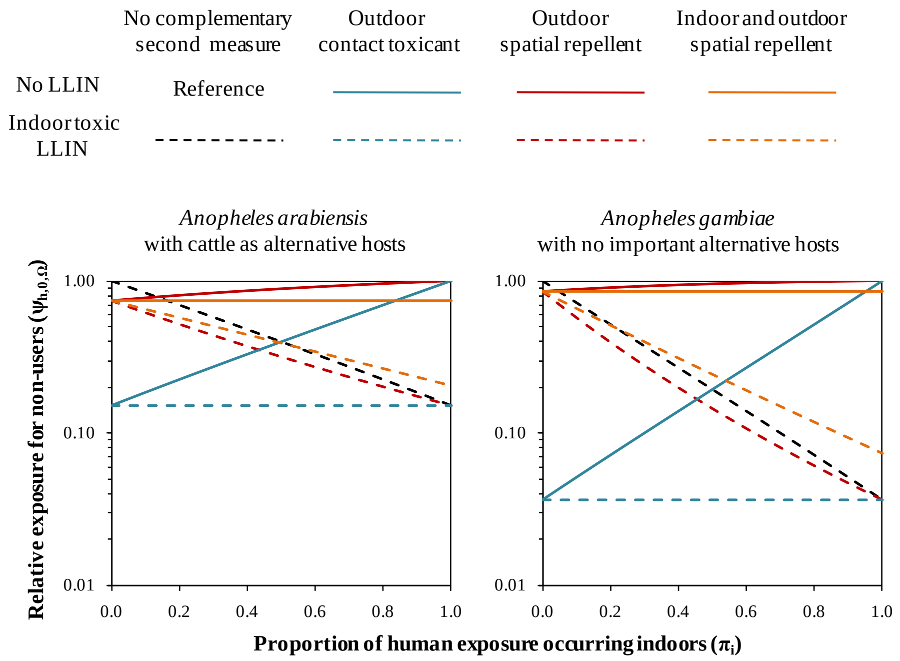

Figure S1. Purely community-level impact of products for outdoor malaria prevention expressed in terms of the mean relative risk of exposure experienced by non-users of any protective measure ($\psi_{h,0,\Omega}$). Scenarios are considered in which LLIN products which provide 50% personal protection (ρ_i = 0·5) by killing half of all mosquitoes that attack them ($\theta_{\mu,pre,i}$ = 0·5) are complemented by use of additional products conferring equivalent personal protection (ρ_o or ρ_{i+o} = 0·5) with one of the three following profiles: Products for exclusively outdoor use which kill attacking mosquitoes before they feed ($\theta_{\mu,pre,o}$ = 0·5) or products which deter mosquitoes from attacking which are used either outdoors only ($\theta_{\Delta,o}$ = 0·5) or are used both indoors and outdoors ($\theta_{\Delta,i+o}$ = 0·5).
