# Supplementary material for: Target product profiles for protecting against outdoor malaria transmission
Source: Malar J. 2012 Jan 11;11:17. doi: 10.1186/1475-2875-11-17 (PMC3298720; doi:10.1186/1475-2875-11-17)
Supplement: Additional file 2 — Figure S2. Additional incremental community-level impact of outdoor contact toxins or repellents that are exclusively used outdoors or used both indoors and outdoors when combined with indoor LLINs with contact toxins, compared with their direct impact as stand-alone intervention strategies. [file 1475-2875-11-17-S2.PDF]

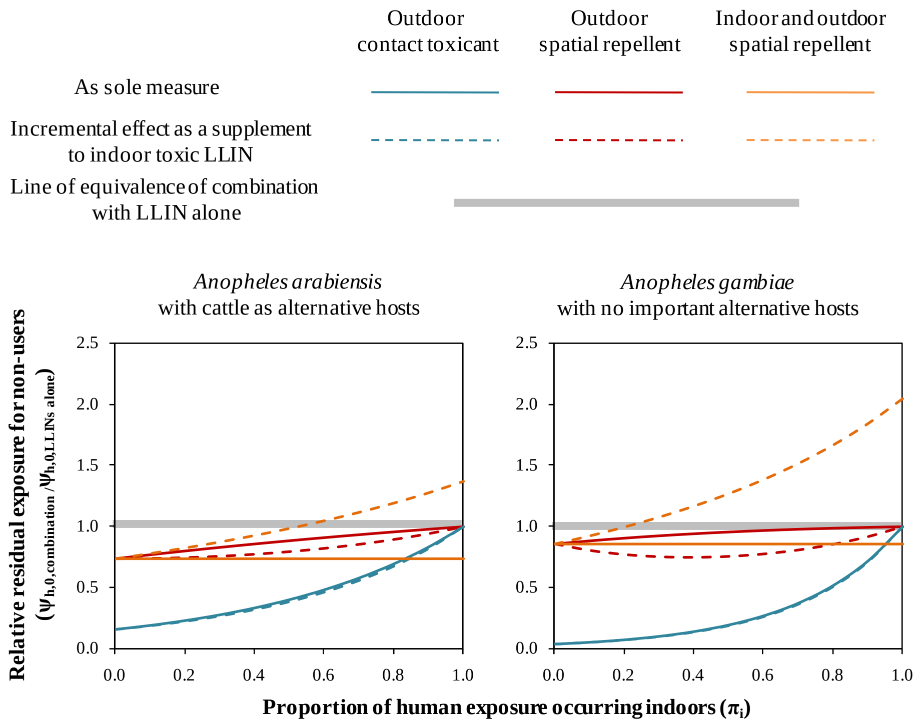

Figure S2. Additional incremental community-level impact of outdoor contact toxins ($\theta_{\mu,pre,o}$ = 0·5) or repellents that are exclusively used outdoors ($\theta_{\Delta,o}$ = 0·5) or used both indoors and outdoors ($\theta_{\Delta,i+o}$ = 0·5) when combined with indoor LLINs with contact toxins ($\theta_{\mu,pre,i}$ = 0·5), compared with their direct impact as stand-alone intervention strategies. Purely community-level impact is expressed in terms of the mean relative risk of exposure to residual transmission for non-users of any protective measure where LLINs are combined with additional products with the above profiles ($\psi_{h,0,combination}$) compared with when they are applied as a stand-alone measure ($\psi_{h,0,LLINs\ alone}$). All products are assumed to confer 50% personal protection (ρ_o or ρ_{i+o} = 0·5) by either repelling or killing half of all mosquitoes that attack them (θ = 0·5).
