# Supplementary material for: Target product profiles for protecting against outdoor malaria transmission
Source: Malar J. 2012 Jan 11;11:17. doi: 10.1186/1475-2875-11-17 (PMC3298720; doi:10.1186/1475-2875-11-17)
Supplement: Additional file 3 — Figure S3. Progressive community-level impact upon a completely outdoor transmission system of products with increasing efficacy of personal protection achieved by either repelling or killing attacking mosquitoes before they feed upon human users. [file 1475-2875-11-17-S3.PDF]

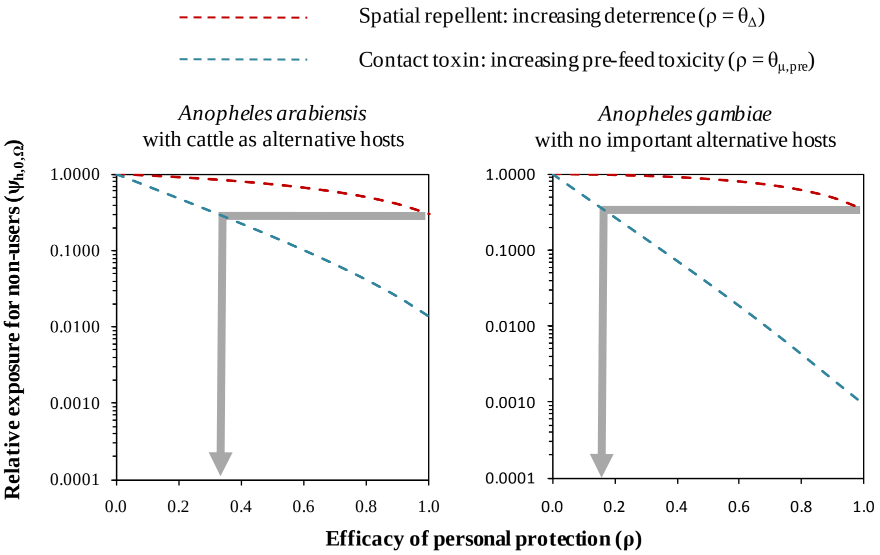

Figure S3. Progressive community-level impact upon a completely outdoor transmission system (π_i = 0) of products with increasing efficacy of personal protection (ρ_o) achieved by either repelling ($\theta_{\Delta,o}$) or killing ($\theta_{\mu,pre,o}$) attacking mosquitoes before they feed upon human users. The grey arrows represent interpolation of the efficacy thresholds at which the toxic mode of action achieves equivalent transmission control to the theoretical limit at complete protective efficacy ($\theta_{\Delta,o} = 1{\cdot}0$ so $\rho_o = 1{\cdot}0$) for spatial repellents at high coverage ($C_h = 0{\cdot}8$). Purely community-level impact is expressed in terms of the mean relative risk of exposure experienced by non-users lacking any protective measure ($\psi_{h,0,\Omega}$).
