# Supplementary material for: Target product profiles for protecting against outdoor malaria transmission
Source: Malar J. 2012 Jan 11;11:17. doi: 10.1186/1475-2875-11-17 (PMC3298720; doi:10.1186/1475-2875-11-17)
Supplement: Additional file 4 — Supplemental methods S4. Detailed model description. [file 1475-2875-11-17-S4.PDF]

Supplemental methods S4: Detailed model description

Overview

Here we extend a family of kinetic models of mosquito behaviour and mortality [1-4], that estimate protection against exposure to malaria in terms of the entomological inoculation rate (EIR) and changes thereof, so that measures to protect against outdoor-biting mosquitoes are also considered. EIR is a proven epidemiological indicator of malaria transmission intensity and a key determinant of disease burden [5, 6], reflecting the number of times an individual is exposed to infectious mosquito bites over a given time period.

The approach described is essentially a behaviourally-explicit extension of existing vector biodemography [7], models, which predict epidemiologically relevant outcomes such as exposure to transmission. The principles and utility of the biodemography–epidemiology models we have adapted [3, 8-10], as well as several others that are based on similar assumptions [11-17], are well established. Notably, this family of models realistically assumes that mosquito behaviour cycles between host seeking, feeding, resting, oviposition-site seeking, oviposition, and back to host seeking again [12]. One notable simplification to keep in mind is that we have assumed complete gonotrophic concordance, meaning that each egg batch requires one and only one blood meal. In reality, the first blood meal typically requires at least one additional pre-gravid blood meal to achieve mature phase II development of the ovaries [18-20] and additional blood meals may even be taken during subsequent gonotrophic cycles [21].

The underlying behavioural and mortality events that determine the input parameters of these biodemographic processes are modelled assuming a "malaria in a bottle" scenario in which populations of identical parasites, vectors, and hosts are mixed homogenously within an enclosed system [22]. We also consider that use of any protective measure does not necessarily protect against mosquito bites at all times or places. Taking the example of LLINs, it is notable that covered individuals only use their nets for approximately one third of a typical day so protection must be assumed to be partial, even for the most nocturnal, indoor-biting vectors, regardless of net efficacy [23]. Such interactions between mosquito and human behaviours are best summarized for indoor interventions, such as LLINs or IRS, in terms of the proportion of human exposure that would otherwise occur indoors (π_i) [23]. Published field estimates of this parameter for malaria vector population from Tanzania and the Solomon Islands indicate that this proportion may fall far short of its optimal maximum value of 1 and may well be dropping in response to increasing selection pressure as ITN coverage increases [23-25]. As recently described [4], we harmonize components of previously published formulations [2, 3, 23, 26] so that this increasingly important *de facto* gap in coverage is treated with far greater clarity and internal consistency.

Conceptualizing host availability

Blood feeding is the most important epidemiological event in the interactions between humans and malaria vector mosquitoes [27]. In this model, the blood acquisition process is considered as having three phases: 1) the mosquito being in a host-seeking state, 2) the mosquito attacking the host (or diverting away) and 3) the mosquito feeding upon the host [3, 10, 28]. This feeding process is considered to be cyclical rather than continuous, so as to more accurately represent natural events [11, 12, 29, 30].

The term *hosts* refers to any vertebrate blood-sources upon which vectors can feed but can also be expanded to include pseudo-hosts such as odour-baited traps even though mosquitoes cannot possibly obtain blood from them [3]. The host-seeking process is considered here as consisting of two successive stages leading to the mosquito attacking the host namely: 1) non-host oriented kinesis, referring to arbitrary movements of the mosquito before it detects host cues, a process which ends with a host encounter event, and 2) host-oriented taxis, referring to directional movements of the mosquito once it encounters and detects the host cues in the environment and starts moving towards the source of those cues, a process which if initiated, either ends with a host attack event, or is aborted resulting in diversion back to kinesis [3].

The original definition of host availability [2, 10, 26, 28] has been adapted to specifically and separately describe the availability of hosts for attack rather than availability of host blood *per se* [3]. Previously, host availability had been described as the product of host encounter rate and feeding probability [2, 10, 26, 28]. Replacing the term *feeding* with the term *attack* allows us to specify that the availability (a) of any host of any species or type (s) for mosquitoes to attack is the product of the rate at which individual vectors encounter that host (ε_s) and the probability that, after this encounter, they will attack the host (γ_s):

$$a_s = \varepsilon_s \gamma_s \quad \text{(Eq. 1)}$$

The correspondingly, the availability of host blood *per se* (z) from a host of any species or type (s) is the product of the encounter rate (ε_s) and the probability that, after this encounter, they will successfully feed upon that particular host (ϕ_s):

$$z_s = \varepsilon_s \phi_s \quad \text{(Eq. 2)}$$

Both of these mean attack and blood availability terms for individual hosts can be multiplied by the respective numbers of each host type (N_s) to obtain total attack or blood availabilities for particular host types (A_s and Z_s, respectively) or for all hosts (A and Z, respectively:

$$A = \sum_s^\infty A_s = \sum_s^\infty a_s N_s \quad \text{(Eq. 3)}$$

$$Z = \sum_s^\infty Z_s = \sum_s^\infty z_s N_s \quad \text{(Eq. 4)}$$

When the mosquito encounters a host, it can either attack the host or it can be diverted from the host. The attack (γ_s) and diversion (Δ_s) probabilities therefore sum to unity.

$$\gamma_s + \Delta_s = 1 \quad \text{(Eq. 5)}$$

After host encounter, all diverted mosquitoes are assumed to re-enter non-host-oriented kinesis afresh. The diversion may include behavioural responses of mosquitoes to non-preferred or protected hosts which prompt them to abort taxis. For preferred hosts, diversion may be induced by defensive behaviour, physical barriers or chemicals used to treat nets or houses, and which repel or irritate mosquitoes.

However, not all vectors that attack the host will successfully feed. To account for mosquitoes that die during this attack process, a term for the mean attack-related mortality (μ_s) is introduced. Previous versions of the model [2, 3, 26] assumed that only two possibilities exist at this stage: either the vector feeds successfully and consequently survives or it dies in the attempt before obtaining a blood meal. Under such assumptions, all mortality risks associated with host attack are expressed as a single mean probability and assumed to occur prior to feeding. The probability of successful feeding per host encounter (ϕ_s) is therefore calculated as follows:

$$\phi_s = \gamma_s (1 - \mu_s) = (1 - \Delta_s)(1 - \mu_s) \quad \text{(Eq. 6)}$$

Coverage, protection and host availability to mosquitoes

Being protected or not is unambiguously defined as being conditional upon both using a protective measure and, more specifically, using it at times when transmission occurs [23]. *De facto* protective coverage of humans ($C_{h,p}$) with a given measure is therefore defined as being the product of crude coverage (C_h) and the proportion of human exposure that occurs at times and places when that measure can be used (π) [23]:

$$C_{h,p} = \pi \, C_h \quad \text{(Eq. 7)}$$

For simplicity, we consider that one measure is available that can protect against malaria vectors only while the user is indoors (i) while another is available that can only be used outdoors (o) so protective coverage of

these two measures ($C_{h,p,i}$ and $C_{h,p,i}$) are delineated into two clearly distinguished exposure compartments with the proportions of human exposure occurring indoors (π_i) and outdoors (π_o) summing to one:

$$\pi_i + \pi_o = 1 \quad \text{(Eq. 8)}$$

$$C_{h,p,i} = \pi_i C_{h,i} = (1 - \pi_o) C_{h,i} \quad \text{(Eq. 9)}$$

$$C_{h,p,o} = \pi_o C_{h,o} = (1 - \pi_i) C_{h,o} \quad \text{(Eq. 10)}$$

At this point, it is also useful to define the crude coverage of various categories of users who are protected by the indoor measure only ($C_{h,i-o}$), the outdoor measure only ($C_{h,o-i}$) or both ($C_{h,o+i}$). These different user categories contribute as follows to the indoor and outdoor factions of protective coverage ($C_{h,p,i}$ and $C_{h,p,o}$):

$$C_{h,i} = C_{h,i-o} + C_{h,i+o} \quad \text{(Eq. 11)}$$

$$C_{h,o} = C_{h,o-i} + C_{h,i+o} \quad \text{(Eq. 12)}$$

The total availability for attack by mosquitoes [3] of protected ($A_{h,p}$) and unprotected humans ($A_{h,u}$) in the community is defined so that individual users that are exposed at times when they do not use them are considered to be unprotected. The total availability of hosts protected against attack while indoors or outdoors is therefore adjusted for this fraction of exposure which is directly preventable by protective measures used in these compartments (π_i and π_o, respectively): The total availabilities for attack of all users at times when they are protected either indoors ($A_{h,p,i}$) or outdoors ($A_{h,p,i}$) are calculated as follows:

$$A_{h,p,i} = a_{h,p,i} N_h \pi_i C_{h,i} = a_{h,p,i} N_h C_{h,p,i} \quad \text{(Eq. 13)}$$

$$A_{h,p,o} = a_{h,p,o} N_h \pi_o C_{h,o} = a_{h,p,o} N_h C_{h,p,o} \quad \text{(Eq. 14)}$$

Where $a_{h,p,i}$ and $a_{h,p,o}$ are the availabilities for attack of an individual human while protected indoors or outdoors, respectively, and N_h is the number of humans present.

The availability of the remaining fraction of humans which are unprotected ($A_{h,u}$) because either they do not use any form of protection or because they are exposed during times when those measures are not used can be calculated as follows where $a_{h,u}$ is the attack availability of an unprotected individual:

$$A_{h,u} = a_{h,u} N_h (1 - C_{h,p,i} - C_{h,p,o}) = a_{h,u} N_h (1 - \pi_i C_{h,i} - \pi_o C_{h,o}) \quad \text{(Eq. 15)}$$

Similarly, to estimate the total availability of blood (Z) from these same categories of human hosts, equivalent formulae based on the availability of blood from individuals protected indoors ($z_{h,p,i}$), those protected outdoors ($z_{h,p,o}$) and those unprotected ($z_{h,u}$) human hosts are applied:

$$Z_{h,p,i} = z_{h,p,i} N_h \pi_i C_{h,i} = z_{h,p,i} N_h C_{h,p,i} \quad \text{(Eq. 16)}$$

$$Z_{h,p,o} = z_{h,p,o} N_h \pi_o C_{h,o} = z_{h,p,o} N_h C_{h,p,o} \quad \text{(Eq. 17)}$$

$$Z_{h,u} = z_{h,u} N_h (1 - C_{h,p,i} - C_{h,p,o}) = z_{h,u} N_h (1 - \pi_i C_{h,i} - \pi_o C_{h,o}) \quad \text{(Eq. 18)}$$

The probabilities of diversion (Δ) and attack related mortality (μ) are considered to be same for cattle (c) and unprotected humans so equation 6 can be specified as follows:

$$\phi_c = \phi_{h,u} = \phi_{h,0} = \gamma_{h,u}(1 - \mu_{h,u}) = (1 - \Delta_{h,u})(1 - \mu_{h,u}) \quad \text{(Eq. 19)}$$

As previously [2-4, 26], mean encounter rates for humans (ε_h) are assumed to be the same for those who are protected and those who are not. Corresponding terms describing the availabilities of protected humans ($a_{h,p,i}$, $a_{h,p,o}$ $z_{h,p,i}$ and $z_{h,p,o}$) can therefore be calculated by modifying the baseline encounter rates, attack probabilities and feeding probabilities as follows. In all cases, where before there were previously single terms

to denote probabilities of diversion (Δ), attack (γ), mortality (μ) and feeding (ϕ), now there is one for hosts encountered and protected indoors (i) and another for those encountered and protected outdoors (o).

$$\gamma_{h,p,i} = 1 - \Delta_{h,p,i} \quad \text{(Eq. 20)}$$

$$\gamma_{h,p,o} = 1 - \Delta_{h,p,o} \quad \text{(Eq. 21)}$$

Where $\Delta_{h,p}$ is the probability that a mosquito will divert away from an encountered, protected human host. However, the effect of attack-related mortality upon the probability of feeding is calculated considering only mortality which occurs before the mosquito feeds ($\mu_{h,p,pre}$):

$$\phi_{h,p,i} = \gamma_{h,p,i}\,(1 - \mu_{h,p,pre,i}) \quad \text{(Eq. 22)}$$

$$\phi_{h,p,o} = \gamma_{h,p,o}\,(1 - \mu_{h,p,pre,o}) \quad \text{(Eq. 23)}$$

Where $\mu_{h,p,pre}$ is the probability that a mosquito will die before feeding if it attacks a protected host.

These terms are calculated as follows based on the probabilities of diversion ($\Delta_{h,u}$) and death ($\mu_{h,u}$) for unprotected humans, combined with the additional probability of diversion (θ_{Δ}) and death before feeding ($\theta_{\mu,pre}$) caused by the deterrent and insecticidal properties of the net:

$$\Delta_{h,p,i} = \Delta_{h,u} + \theta_{\Delta,\mathrm{i}}\,(1 - \Delta_{h,u}) \quad \text{(Eq. 24)}$$

$$\Delta_{h,p,o} = \Delta_{h,u} + \theta_{\Delta,\mathrm{o}}\,(1 - \Delta_{h,u}) \quad \text{(Eq. 25)}$$

And $$\mu_{h,p,pre,i} = \mu_{h,u} + \theta_{\mu,pre,i}\,(1 - \mu_{h,u}) \quad \text{(Eq. 26)}$$

$$\mu_{h,p,pre,o} = \mu_{h,u} + \theta_{\mu,pre,o}\,(1 - \mu_{h,u}) \quad \text{(Eq. 27)}$$

This distinction between fast- and slow-acting insecticidal activities necessitates that the total excess attack-related mosquito mortality resulting from using an LLIN (θ_{μ}) is specified as the sum of the excess mortality which occurs before ($\theta_{\mu,pre}$) or after ($\theta_{\mu,post}$) obtaining a bloodmeal:

$$\theta_{\mu,i} = \theta_{\mu,pre,i} + \theta_{\mu,post,i} \quad \text{(Eq. 28)}$$

$$\theta_{\mu,o} = \theta_{\mu,pre,o} + \theta_{\mu,post,o} \quad \text{(Eq. 29)}$$

While insecticide-related mosquito mortality occurring after the mosquito has fed on the protected host does not contribute to personal protection, it does contribute to community-level suppression of malaria transmission by reducing population mean mosquito survival. The term $\mu_{h,p}$ is therefore calculated separately as follows:

$$\mu_{h,p,i} = \mu_{h,u} + \theta_{\mu,i}\,(1 - \mu_{h,u}) \quad \text{(Eq. 30)}$$

$$\mu_{h,p,o} = \mu_{h,u} + \theta_{\mu,o}\,(1 - \mu_{h,u}) \quad \text{(Eq. 31)}$$

Mosquito population parameters

This distinction between killing mosquitoes before or after feeding on the protected host allows the proportion of bloodmeals derived from humans (Q_h) to be calculated as previously described [4] except that distinct availability terms for those protected indoors and outdoors are specified:

$$Q_h = \frac{z_{h,u}+z_{h,p,i}+z_{h,p,o}}{z} \quad \text{(Eq. 32)}$$

The feeding cycle length (f) is calculated as the sum of the durations of the gestation period (g), the oviposition site-seeking interval (η_v) and the vertebrate host-seeking interval (η_v):

$$f = g + \eta_o + \eta_v \quad \text{(Eq. 33)}$$

Survival across all phases of the gonotrophic cycle is calculated as the distinct daily survival probability during each phase to the power of the respective time intervals, namely the host-seeking interval (η_v), gestation period (g) and oviposition site-seeking interval (η_o). The daily survival probability of a resting mosquito is defined as P and the survival probabilities during host-seeking and oviposition site-seeking are assumed to be equal and are both defined using the term P_{ov}. The survival rate per feeding cycle (P_f) was therefore estimated as the combined probability that a vector survives gestation (P^g), oviposition site-seeking ($P_{ov}^{\eta_o}$), vertebrate host-seeking ($P_{ov}^{\eta_v}$) and the eventual attack of a host ($P_{ov}^{\eta_v+\eta_o}$):

$$P_f = P^g \, P_{ov}^{\eta_v} \, P_{ov}^{\eta_o} \, P_\gamma = P^g \, P_{ov}^{\eta_o+\eta_v} \, P_\gamma \quad \text{(Eq. 34)}$$

The probability of surviving host attack per feeding cycle (P_γ) is also calculated as previously described [3] except that the attack availability of humans protected indoors and outdoors are distinguished

$$P_\gamma = 1 - \left(\frac{\mu_{h,u} \, A_{h,u} + \mu_{h,p,i} \, A_{h,p,i} + \mu_{h,p,o} \, A_{h,p,o} + \mu_c \, A_c}{A_{h,u} + A_{h,p,i} + A_{h,p,o} + A_c}\right) \quad \text{(Eq. 35)}$$

The mean seeking interval for vertebrate hosts (η_v) can be calculated as the reciprocal of total host availability (A), using estimates of these feeding probabilities and their corresponding encounter rates:

$$\eta_v = 1/A = 1/(A_{h,u} + A_{h,p,i} + A_{h,p,o} + A_c) \quad \text{(Eq. 36)}$$

The oviposition site-seeking interval (η_o) is correspondingly calculated based on the expectation that mosquitoes forces to fly further and longer in search of blood with have to also fly proportionally further and longer in search of oviposition sites once the blood meal has been digested and eggs are matured. This term is therefore calculated as the reciprocal of total availability of utilizable aquatic habitat (A_a) [10]:

$$\eta_o = 1/A_a \quad \text{(Eq. 37)}$$

Note that the equivalent equation for calculating in the most recently published formulation of this model [4] mistakenly describes η_o as depending upon total availability of aquatic habitat in terms of total utilization rate (Z) rather than approach rate (A). Note however, that this makes no practical difference to the simulated outcomes as the baseline values set for this parameter remain unchanged.

Total aquatic habitat availability (A_a) is assumed to vary proportionally with vertebrate blood availability (Z) as it changes from baseline (0) to intervention (Ω) scenarios, reflecting the intrinsically endogenous relationship between host and aquatic habitat availability as discussed in detail elsewhere [4]:

$$Z_{a,\Omega} = Z_{a,0} \, Z_\Omega / Z_0 \quad \text{(Eq. 38)}$$

The biodemography component of the model is adapted to a daily cycle and cumulative survival up to each age (x) is estimated as follows [2]:

$$P_x = P_f^{x/f} \quad \text{(Eq. 39)}$$

Similarly, the sporozoite infection prevalence of mosquitoes at each age is considered in days, accounting for superinfection:

$$S_x = S_{x-1} + \frac{\kappa \, Q_h (1 - S_{x-1})}{f} \text{ where } x > n \text{ otherwise } S_x = 0 \quad \text{(Eq. 40)}$$

where κ denotes the mean infectiousness of the human population to vector mosquitoes [31] and n is the duration of the sporogonic development period of the parasite from ingestion to infective sporozoite stages

[9]: Survival and infectveness probabilities are calculated up to 40 days, after which the contributions of mosquitoes in these age classes to transmission become negligible. Note that P_x is multiplied by S_x to obtain the corresponding probability of being both alive and infective (I_x) on each day

The following mosquito lifetime biodemographic parameters are calculated by summing these three age-specific outcomes as previously described [2, 9]. The number of human bites the average mosquito takes in a lifetime (b_h) is defined as the sum of the probabilities of surviving and feeding on a human at each age (x):

$$b_h = \frac{Q_h}{f} \sum_x^\infty P_x \qquad \text{(Eq. 41)}$$

Note that to enable incorporation of survival-dependent emergence rates, we also similarly calculate the number of human bites on all hosts, rather than just humans, per mosquito lifetime (b):

$$b = \frac{1}{f} \sum_x^\infty P_x \qquad \text{(Eq. 42)}$$

Accounting for superinfection, the number of infectious bites on humans per mosquito lifetime (β_h) is calculated as the product of the human blood index and sum of the products of the probabilities of biting and being infectious at each age [2, 9]:

$$\beta_h = \frac{Q_h}{f} \sum_x^\infty S_x P_x \qquad \text{(Eq. 43)}$$

Again, the number of sporozoite-infected bites on all hosts per mosquito lifetime (β), regardless of whether that host is susceptible to infection or not, is calculated similarly but ignoring the human blood index term:

$$\beta = \frac{1}{f} \sum_x^\infty S_x P_x \qquad \text{(Eq. 44)}$$

The overall sporozoite prevalence in the vector population (S) can then be calculated as β_h divided by b_h:

$$S = \beta_h / b_h = \beta / b \qquad \text{(Eq. 45)}$$

Emergence rate was assumed to vary simply and linearly with mean number of successfully-completed feeding cycles by adult mosquitoes (b), calculated as described in equation 42. Emergence rate in a given vector control scenario (E_Ω) was therefore calculated as the product of the maximum emergence rate expected in the absence of any adult mosquito control (E_0) and the relative value of the mean number of feeding cycles per mosquito lifetime in that scenario (b_Ω), compared with such baseline conditions (b_0):

$$E_\Omega = E_0 b_\Omega / b_0 \qquad \text{(Eq. 46)}$$

Epidemiological outcomes: dealing with partially covered, partially protected humans

The entomologic inoculation rate (EIR) for non-users ($EIR_{h,0}$) can be directly estimated based on the share of all available blood sources which a single non-user represents ($z_{h,u}/Z$) multiplied by the total number of infectious bites on all hosts (β) by all emerging mosquitoes (E):

$$EIR_{h,0} = \frac{z_{h,u}\, \beta\, E}{Z} \qquad \text{(Eq. 47)}$$

Alternatively, this parameter may be estimated by considering only infectious bites on human hosts (β_h) and therefore considering only the share of available human blood which such an individual represents:

$$EIR_{h,0} = \frac{z_{h,u}\, \beta_h\, E}{Z_h} \qquad \text{(Eq. 48)}$$

Nevertheless, it is essential to retain the protection-weighted mean terms for parameters which reflect the properties of individual net users who are only covered with the protective LLIN for proportion of their

normal exposure (π_i) and uncovered and unprotected for the remained ($1-\pi_i$). We therefore retain these terms but annotate them more distinctly than previously [26] so that the attack probability ($\gamma_{h,net}$ rather than $\bar{\gamma}_{h,p}$) and feeding probability ($\phi_{h,net}$ rather than $\bar{\phi}_{h,p}$) reflect the mean of protected and unprotected periods for net users but cannot be confused with the corresponding probabilities for net users during the specific periods when they are protected ($\gamma_{h,p}$ and $\phi_{h,p}$, respectively).

$$\gamma_{h,i-o} = \pi_i\, \gamma_{h,p,i} + (1-\pi_i)\gamma_{h,u} \quad \text{(Eq. 49)}$$

$$\gamma_{h,o-i} = \pi_i\, \gamma_{h,u} + (1-\pi_i)\gamma_{h,p,o} \quad \text{(Eq. 50)}$$

$$\gamma_{h,i+o} = \pi_i\, \gamma_{h,p,i} + (1-\pi_i)\gamma_{h,p,o} \quad \text{(Eq. 51)}$$

$$\phi_{h,i-o} = \pi_i\, \phi_{h,p,i} + (1-\pi_i)\phi_{h,u} \quad \text{(Eq. 52)}$$

$$\phi_{h,o-i} = \pi_i\, \phi_{h,u} + (1-\pi_i)\phi_{h,p,o} \quad \text{(Eq. 53)}$$

$$\phi_{h,i+o} = \pi_i\, \phi_{h,p,i} + (1-\pi_i)\phi_{h,p,o} \quad \text{(Eq. 54)}$$

Derived terms such as attack availability ($a_{h,p,i-o}$, $a_{h,p,o-i}$ and $a_{h,p,i+o}$) and blood availability ($z_{h,p,i-o}$, $z_{h,p,o-i}$ and $z_{h,p,i+o}$), as well as corresponding terms for relative attack availability ($\lambda_{h,p,i-o}$, $\lambda_{h,p,o-i}$ and $\lambda_{h,p,i+o}$) and exposure to bites ($\psi_{h,p,i-o}$, $\psi_{h,p,o-i}$ and $\psi_{h,p,i+o}$) compared with non-users, can be calculated as previously described.

$$a_{h,p,i-o} = \varepsilon_h\, \gamma_{h,i-o} \quad \text{(Eq. 55)}$$

$$a_{h,p,o-i} = \varepsilon_h\, \gamma_{h,o-i} \quad \text{(Eq. 56)}$$

$$a_{h,p,i+o} = \varepsilon_h\, \gamma_{h,i+o} \quad \text{(Eq. 57)}$$

$$z_{h,p,i-o} = \varepsilon_h\, \phi_{h,p,i-o} \quad \text{(Eq. 58)}$$

$$z_{h,p,o-i} = \varepsilon_h\, \phi_{h,p,o-i} \quad \text{(Eq. 59)}$$

$$z_{h,p,i+o} = \varepsilon_h\, \phi_{h,p,i+o} \quad \text{(Eq. 60)}$$

$$\lambda_{h,p,i-o} = \frac{a_{h,p,i-o}}{a_{h,0}} \quad \text{(Eq. 61)}$$

$$\lambda_{h,p,o-i} = \frac{a_{h,p,o-i}}{a_{h,0}} \quad \text{(Eq. 62)}$$

$$\lambda_{h,p,i+o} = \frac{a_{h,p,i+o}}{a_{h,0}} \quad \text{(Eq. 63)}$$

$$\psi_{h,p,i-o} = \frac{z_{h,p,i-o}}{z_{h,0}} \quad \text{(Eq. 64)}$$

$$\psi_{h,p,o-i} = \frac{z_{h,p,o-i}}{z_{h,0}} \quad \text{(Eq. 65)}$$

$$\psi_{h,p,i+o} = \frac{z_{h,i+o}}{z_{h,0}} \quad \text{(Eq. 66)}$$

The EIR experienced by various user categories can be calculated as follows:

$$EIR_{h,p,i-o} = \frac{z_{h,p,i-o}\,\beta\, E}{Z} = \psi_{h,p,i-o} EIR_{h,0} \quad \text{(Eq. 67)}$$

$$EIR_{h,p,o-i} = \frac{z_{h,p,o-i}\,\beta\, E}{Z} = \psi_{h,p,o-i} EIR_{h,0} \quad \text{(Eq. 68)}$$

$$EIR_{h,p,i+o} = \frac{z_{h,p,i+o}\,\beta\, E}{z} = \psi_{h,p,i+o} EIR_{h,0} \quad \text{(Eq. 69)}$$

The mean EIR experienced in scenario Ω by the mixture of non-users and various user categories which comprise the community ($\psi_{h,\Omega}$) can be independently calculated in two distinct and independent by either simply weighting the EIR parameters for all user and non-user categories according to crude coverage and the gap in coverage, respectively:

$$EIR_{h,\Omega} = C_{h,i-o} EIR_{h,i-o,\Omega} + C_{h,o-i} EIR_{h,o-i,\Omega} + C_{h,i+o} EIR_{h,i+o,\Omega} + \left(1 - C_{h,i-o} - C_{h,o+i} - C_{h,i+o}\right) EIR_{h,0,\Omega} \quad \text{(Eq.70)}$$

This community mean EIR can also be calculated with a simpler formula derived from first principles [9]:

$$EIR_{h,\Omega} = \frac{\beta_h\, E}{N_h} \quad \text{(Eq. 71)}$$

Similarly, the relative exposure of non-users and all user categories ($\psi_{h,0,\Omega}$ and $\psi_{h,net,\Omega}$) and community-wide mean relative exposure ($\psi_{h,\Omega}$) in a given intervention scenario (Ω) is calculated using the terms $EIR_{h,0,\Omega}$ and $EIR_{h,0,0}$ to denote the EIR experienced by non-users in a scenario with and without interventions in place, respectively, while $EIR_{h,i-o,\Omega}$, $EIR_{h,o-i,\Omega}$ and $EIR_{h,i+o,\Omega}$ represent that experienced by each corresponding user category under intervention scenario Ω:

$$\psi_{h,0,\Omega} = \frac{EIR_{h,0,\Omega}}{EIR_{h,0,0}} \quad \text{(Eq. 72)}$$

$$\psi_{h,i-o,\Omega} = \frac{EIR_{h,i-o,\Omega}}{EIR_{h,0,0}} \quad \text{(Eq. 73)}$$

$$\psi_{h,o-i,\Omega} = \frac{EIR_{h,o-i,\Omega}}{EIR_{h,0,0}} \quad \text{(Eq. 74)}$$

$$\psi_{h,i+o,\Omega} = \frac{EIR_{h,i+o,\Omega}}{EIR_{h,0,0}} \quad \text{(Eq. 75)}$$

$$\psi_{h,\Omega} = \frac{EIR_{h,\Omega}}{EIR_{h,0,0}} \quad \text{(Eq. 76)}$$

Parameterization of the model

The parameters of the model were set exactly as previously described [4] with the following adaptations. The term π_i is tuned across its full range of possible values from 0, reflecting a completely exophagic mosquito population and/or a human population that sleeps entirely outdoors, to 1, reflecting a purely endophagic vector population and/or a human population that never leaves their houses during hours of mosquito activity. The daily survival rate for mosquitoes foraging for vertebrate blood sources or for oviposition sites (P_{ov}) was set at 0.85 as previously described and justified based on sensitivity analysis [4].

All other parameter settings for the two vector population scenarios (*Anopheles arabiensis* representing a mosquito that can exploit non-human hosts compared with *An. gambiae* which is almost exclusively dependent on humans for blood) are as previously described for a village with 1000 people and an equal number of cattle [3, 4]. Consistent with previous simulations [3, 4], the maximum emergence rate of mosquitoes in the absence of adult mosquito control measures (E_0) was set at 2×10^7 adult mosquitoes per year.

While this model can just as readily simulate the impact of IRS, only LLINs were considered as a potential means of indoor protection because the combination of the physical barrier or the net and the fast-acting toxicity of their pyrethroid active ingredients allow them to be directly compared with spatial repellents or insecticidal clothing that also confer direct personal or household protection [32]. By comparison, many IRS formulations are relatively slow-acting, usually killing mosquitoes after they have fed, so that the comparison

between repellent and toxic modes of action is confounded by the differences between slow and fast-acting toxins [4, 32]. Therefore, no scenarios including IRS as the indoor protection measure were simulated. In all simulated intervention scenarios, crude coverage of humans for a given intervention was set at 80% ($C_h = 0.8$) in line with the Roll Back Malaria targets for coverage of all age groups with LLINs and IRS [33] which represents an ambitious but realistically achievable target for most malaria-afflicted developing nations. Coverage of individuals with either of two interventions intended to cover indoor and outdoor exposure compartments ($C_{h,i} = 0.8$, $C_{h,o} = 0.8$) was assumed to occur independently and randomly ($C_{h,i-o} = 0.16$, $C_{h,o-i} = 0.16$, $C_{h,i+o} = 0.64$). In the case where the purely spatial repellent product intended for outdoor use ($\theta_{\Delta,o} = 0.5$, $\theta_{\mu,i,pre} = 0$, $\theta_{\mu,i,post} = 0$) was also used indoors where it could interact with indoor LLINs with purely fast-acting contact insecticidal properties ($\theta_{\Delta,o} = 0$, $\theta_{\mu,i,pre} = 0.5$, $\theta_{\mu,i,post} = 0$), this combination of properties was also assumed to occur randomly and independently, and was incorporated by adding spatial repellent properties to the indoor intervention as previously described,[4] in direct proportion to coverage ($\theta_{\Delta,o} = 0.4$, $\theta_{\mu,i,pre} = 0.5$, $\theta_{\mu,i,post} = 0$).

References

1. Killeen GF, Kihonda J, Lyimo E, Okech FR, Kotas ME, Mathenge E, Schellenberg J, Lengeler C, Smith TA, Drakeley C: **Quantifying behavioural interactions between humans and mosquitoes: Evaluating the protective efficacy of insecticidal nets against malaria transmission in rural Tanzania.** *BMC Infect Dis* 2006, **6:** 161.
2. Killeen GF, Smith TA: **Exploring the contributions of bednets, cattle, insecticides and excito-repellency to malaria control: A deterministic model of mosquito host-seeking behaviour and mortality.** *Trans R Soc Trop Med Hyg* 2007, **101:** 867-880.
3. Okumu FO, Moore SJ, Govella NJ, Chitnis N, Killeen GF: **Potential benefits, limitations and target product-profiles of odor-baited mosquito traps as a means of malaria control.** *PLoS One* 2010, **5:** e11573.
4. Killeen GF, Chitnis N, Moore SJ, Okumu FO: **Target product profile choices for intra-domiciliary malaria vector control pesticide products: repel or kill?** *Malar J* 2011, **10:** 207.
5. Smith TA, Leuenberger R, Lengeler C: **Child mortality and malaria transmission intensity in Africa.** *Trends Parasitol* 2001, **17:** 145-149.
6. Smith DL, Dushoff J, Snow RW, Hay SI: **The entomological inoculation rate and *Plasmodium falciparum* infection in African children.** *Nature* 2005, **438:** 492-495.
7. Carey JR: **Insect biodemography.** *Annu Rev Entomol* 2001, **46:** 79-110.
8. Killeen GF, McKenzie FE, Foy BD, Schieffelin C, Billingsley PF, Beier JC: **The potential impacts of integrated malaria transmission control on entomologic inoculation rate in highly endemic areas.** *Am J Trop Med Hyg* 2000, **62:** 545-551.
9. Killeen GF, McKenzie FE, Foy BD, Schieffelin C, Billingsley PF, Beier JC: **A simplified model for predicting malaria entomologic inoculation rates based on entomologic and parasitologic parameters relevant to control.** *Am J Trop Med Hyg* 2000, **62:** 535-544.
10. Killeen GF, Seyoum A, Knols BGJ: **Rationalizing historical successes of malaria control in Africa in terms of mosquito resource availability management.** *Am J Trop Med Hyg* 2004, **71 (Supplement 2):** 87-93.
11. Saul A: **Zooprophylaxis or zoopotentiation: the outcome of introducing animals on vector transmission is highly dependent on the mosquito mortality while searching.** *Malar J* 2003, **2:** 32.
12. Saul AJ, Graves PM, Kay BH: **A cyclical feeding model for pathogen transmission and its application to determine vectorial capacity from vector infection rates.** *J Appl Ecol* 1990, **27:** 123-133.
13. Smith DL, McKenzie FE: **Statics and dynamics of malaria infection in Anopheles mosquitoes.** *Malar J* 2004, **3:** 13.

14. Griffin JT, Hollingsworth TD, Okell LC, Churcher TS, White M, Hinsley W, Bousema T, Drakeley CJ, Ferguson NM, Basáñez MG, Ghani AC: **Strategies towards *Plasmodium falciparum* malaria elimination in Africa using currently available tools.** *PLoS Med* 2010, **7:** e1000324.
15. Smith DL, McKenzie FE, Snow RW, Hay SI: **Revisiting the basic reproductive number for malaria and its implications for malaria control.** *PLoS Biol* 2007, **5:** e42.
16. Chitnis N, Schapira A, Smith T, Steketee R: **Comparing the effectiveness of malaria vector-control interventions through a mathematical model.** *Am J Trop Med Hyg* 2010, **83:** 230-240.
17. Yakob L, Dunning R, Yan G: **Indoor residual spray and insecticide-treated bednets for malaria control: theoretical synergisms and antagonisms.** *J R Soc Interface* 2010, **8:** 799-806.
18. Gillies MT: **A modified technique for age grading populations of *Anopheles gambiae*.** *Ann Trop Med Parasitol* 1958, **58:** 261-273.
19. Gillies MT: **Studies on the dispersion and survival of *Anopheles gambiae* in East Africa, by means of marking and release experiments.** *Bull Entomol Res* 1961, **52:** 99-127.
20. Gillies MT, Wilkes TJ: **A study of the age-composition of populations of *Anopheles gambiae* Giles and *A. funestus* Giles in North-Eastern Tanzania.** *Bull Entomol Res* 1965, **56:** 237-262.
21. Beier JC: **Frequent blood-feeding and restrictive sugar-feeding behavior enhance the malaria vector potential of *Anopheles gambiae s.l.* and *An. funestus* (Diptera:Culicidae) in western Kenya.** *J Med Entomol* 1996, **33:** 613-618.
22. Killeen GF, Knols BG, Gu W: **Taking malaria transmission out of the bottle: implications of mosquito dispersal for vector-control interventions.** *Lancet Infect Dis* 2003, **3:** 297-303.
23. Govella NJ, Okumu FO, Killeen GF: **Insecticide-treated nets can reduce malaria transmission by mosquitoes which feed outdoors.** *Am J Trop Med Hyg* 2010, **82:** 415-419.
24. Russell TL, Govella NJ, Azizi S, Drakeley CJ, Kachur SP, Killeen GF: **Increased proportions of outdoor feeding among residual malaria vector populations following increased use of insecticide-treated nets in rural Tanzania.** *Malar J* 2011, **10:** 80.
25. Bugoro H, Cooper RD, Butafa C, Iro'ofa C, Mackenzie DO, Chen C-C, Russell TL: **Bionomics of the malaria vector *Anopheles farauti* in Temotu Province, Solomon Islands: issues for malaria elimination.** *Malar J* 2011, **10:** 133.
26. Killeen GF, Smith TA, Ferguson HM, Abdulla S, Mshinda H, Lengeler C, Kachur SP: **Preventing childhood malaria in Africa by protecting adults from mosquitoes with insecticide-treated nets.** *PLoS Med* 2007, **4:** e229.
27. MacDonald G: *The epidemiology and control of malaria.* London: Oxford University Press; 1957.
28. Killeen GF, McKenzie FE, Foy BD, Bogh C, Beier JC: **The availability of potential hosts as a determinant of feeding behaviours and malaria transmission by mosquito populations.** *Trans R Soc Trop Med Hyg* 2001, **95:** 469-476.
29. Chitnis N, Smith TA, Steketee R: **A mathematical model for the dynamics of malaria in mosquitoes feeding on a heterogeneous host population.** *J Biol Dynamics* 2008, **2:** 259-285.
30. Le Menach A, Takala S, McKenzie FE, Perisse A, Harris A, Flahault A, Smith DL: **An elaborated feeding cycle model for reductions in vectorial capacity of night-biting mosquitoes by insecticide-treated nets.** *Malar J* 2007, **6:** 10.
31. Killeen GF, Ross A, Smith TA: **Infectiousness of malaria-endemic human populations to vector mosquitoes.** *Am J Trop Med Hyg* 2006, **76 (Suppl. 2):** 38-45.
32. Okumu FO, Moore SJ: **Combining indoor residual spraying and insecticide-treated nets for malaria control in Africa: a review of possible outcomes and an outline of suggestions for the future.** *Malar J* 2011, **10:** 208.
33. WHO: **Insecticide treated mosquito nets: A position statement.** Geneva: Global Malaria Programme; World Health Organization; 2007: pp. 10.
34. Korenromp EL, Miller J, Cibulskis RE, Kabir Cham M, Alnwick D, Dye C: **Monitoring mosquito net coverage for malaria control in Africa: possession vs. use by children under 5 years.** *Trop Med Int Health* 2003, **8:** 693-703.
35. Miller JM, Korenromp EL, Nahlen BL, R WS: **Estimating the number of insecticide-treated nets required by African households to reach continent-wide malaria coverage targets.** *JAMA* 2007, **297:** 2241-2250.

36. Roberts DR, Alecrim WD, Hshieh P, Grieco JP, Bangs M, Andre RG, Chareonviriphap T: **A probability model of vector behavior: effects of DDT repellency, irritancy, and toxicity in malaria control.** *J Vector Ecol* 2000, **25:** 48-61.
37. Beier JC, Killeen GF, Githure J: **Short report: Entomologic inoculation rates and *Plasmodium falciparum* malaria prevalence in Africa.** *Am J Trop Med Hyg* 1999, **61:** 109-113.
38. Hay SI, Rogers DJ, Toomer JF, Snow RW: **Annual *Plasmodium falciparum* entomological inoculation rates across Africa: literature survey, internet access and review.** *Trans R Soc Trop Med Hyg* 2000, **94:** 113-127.
39. Smith TA, Maire N, Dietz K, Killeen GF, Vounatsou P, Molineaux L, Tanner M: **Relationship between entomologic inoculation rate and the force of infection for *Plasmodium falciparum* malaria.** *Am J Trop Med Hyg* 2006, **75 (Supplement 2):** 11-18.
40. Kouznetsov RL: **Malaria control by application of indoor spraying of residual insecticides in tropical Africa and its impact on community health.** *Tropical Doctor* 1977, **7:** 81-93.
41. Pluess B, Tanser FC, Lengeler C, Sharp BL: **Indoor residual spraying for preventing malaria.** *Cochrane Database of Systematic Reviews* 2010, **4:** CD006657.
42. Ross A, Killeen GF, Smith TA: **Relationships of host infectivity to mosquitoes and asexual parasite density in *Plasmodium falciparum*.** *Am J Trop Med Hyg* 2006, **75 (Suppl. 2):** 32-37.
43. Guillet P, Alnwick D, Cham MK, Neira M, Zim M, Heymann D, Mukelebai K: **Long-lasting treated mosquito nets: A breakthrough in malaria prevention.** *Bull World Health Organ* 2001, **79:** 998.
44. Gillies MT: **Studies in house-leaving and outside resting of *Anopheles gambiae* Giles and *Anopheles funestus* Giles in East Africa.** *Bull Entomol Res* 1954, **45:** 375-387.

Table S1: Definitions and explanations for symbols and abbreviations.

Symbol	Definition and explanation	References
a	Availability of individual hosts for attack: rate at which a single mosquito encounters and then attacks a given single host or pseudo-host.	3, 4
A	Total availability of hosts and pseudo hosts: rate at which a single mosquito encounters and attacks all hosts and pseudo hosts.	3, 4
A_a	Total availability of aquatic habitats: rate at which a single mosquito encounters and successfully oviposits into all aquatic habitats	10
b_h	The mean number of bites upon humans per emerging mosquito during its lifetime.	2, 4, 9
b	The mean number of bites upon all human and non-human hosts per emerging mosquito during its lifetime.	This paper
β_h	The mean number of infectious, sporozoite-infected bites upon humans per emerging mosquito during its lifetime.	2, 4, 9
β	The mean number of sporozoite-infected bites upon all hosts, regardless of their susceptibility to infection, per emerging mosquito during its lifetime.	4
c	Cattle.	2-4, 10, 26, 28
C_h	Crude coverage: Proportion of people using LLIN as estimated in standardized malaria indicator surveys [34, 35].	2-4, 10, 26, 28
$C_{h,p,i}$ or $C_{h,p,o}$	Protective coverage: The proportion of all exposure of the human population which is effectively covered by use of measures which protect against indoor or outdoor transmission respectively.	This paper
DDT	Dichloro-diphenyl-dicloroethylene	36
Δ	Probability that a mosquito which encounters a host will be diverted from that host.	2-4, 26
ε	Host-encounter rate: rate at which a single host-seeking mosquito encounters a given single hosts.	2-4, 10, 26, 28
E	Emergence rate of mosquito vectors per year.	2-4, 10, 26
EIR	Entomological inoculation rate (mean number of infectious bites that an average individual human receives per year).	6, 37-39
ϕ	Probability that a mosquito which attacks a host will successfully feed upon that host.	2-4, 10, 26, 28
f	Feeding cycle length: measured as the number of days it takes a single mosquito to get from one blood feed to the next.	2-4, 10, 26
g	Gestation interval: number of days a mosquito takes to digest a blood meal and return to searching for oviposition site.	2-4, 10, 26

h or c	Humans or cattle, respectively.	2-4, 10, 26, 28
i or o	Indoor and outdoor exposure compartments and corresponding intervention use and protection	This paper
$0, i-o, o-i,$ or $i+o$	Users of no protective measure, indoor measure only, outdoor measure or both indoor and outdoor measures, respectively	This paper
IRS	Indoor residual spraying	40, 41
κ	Human infectiousness to mosquitoes: probability of a vector becoming infected per human bite.	9, 12, 31, 42
$LLIN$	Long-lasting insecticidal net	43
λ	Relative availability for attack of a given non-human host type, calculated as quotient of the mean individual attack availability of those hosts divided by the mean individual attack availability of humans not using LLINs.	3, 4
μ	Probability that a mosquito which attacks a host will die during the attack.	2-4, 26
η_o	Oviposition site-seeking interval: number of days that a mosquito takes to find an oviposition site once it starts searching for it.	2-4, 10, 26
η_v	Host-seeking interval: number of days a mosquito takes to find and attack a vertebrate host.	2-4, 10, 26
N	Number of hosts.	2-4, 10, 26
θ_Δ	Excess proportion of mosquitoes which are diverted while attempting to attack a human while that person is using an LLIN.	3, 4
θ_μ	Excess proportion of mosquitoes which are killed while attacking a human while that person is using an LLIN.	3, 4
$\theta_{\mu,pre}$	Excess proportion of mosquitoes which are killed before blood feeding while attacking a human while that person is using an LLIN.	4
$\theta_{\mu,post}$	Excess proportion of mosquitoes which are killed after blood feeding while attacking a human while that person is using an LLIN.	4
Ω or 0	Intervention package scenarios consisting of a specific coverage with LLINs with specific deterrent and toxic properties, with 0 denoting baseline conditions with negligible net coverage, simulated by setting C_h=0.001	3, 4
π_i or π_o	The proportion of normal exposure to mosquito bites upon humans lacking LLINs, which occurs either indoors or outdoors.	This paper
p or u	Specifies values of parameters for humans while actually using and protected by an LLIN, or those which are unprotected who do not use or are outside of their nets, respectively.	This paper
P	Probability that a resting mosquito survives any one day.	2, 4, 44

P_f	Probability that a mosquito survives a single complete feeding cycle.	2-4, 9, 10, 26
P_{ov}	Probability that a mosquito survives any full day of the oviposition site-seeking interval or host-seeking interval.	2-4, 26
Q_h	Human blood index: the proportion of all blood meals from all hosts which are obtained from humans.	2-4, 9, 10, 26
γ	Probability that a mosquito attacks an encountered host.	2-4, 26
ψ	Relative exposure of different hosts other than unprotected humans to infectious mosquito bites: calculated as a ratio of exposure of those hosts to exposure of humans not using nets.	3, 4
WHO	World Health Organization	
z	Availability of blood from an individual host: rate at which a single mosquito encounters, attacks and successfully feeds upon a given single host	3, 4
Z, Z_h, Z_c	Total availability of blood from all hosts, all humans and all cattle, respectively: rate at which a single mosquito encounters, attacks and successfully feeds upon these host sets	3, 4
